# Dihydropyridines Potentiate ATP-Induced Currents Mediated by the Full-Length Human P2X5 Receptor

**DOI:** 10.3390/molecules27061846

**Published:** 2022-03-11

**Authors:** Ida C. Schiller, Kenneth A. Jacobson, Zhiwei Wen, Aparna Malisetty, Günther Schmalzing, Fritz Markwardt

**Affiliations:** 1 Julius-Bernstein-Institute for Physiology, Martin-Luther-University Halle-Wittenberg, Magdeburger Str. 6, 06097 Halle, Germany; ida.ch.schiller@gmail.com; 2 Laboratory of Bioorganic Chemistry & Molecular Recognition Section, National Institute of Diabetes & Digestive & Kidney Diseases, National Institutes of Health, Bethesda, MD 20892, USA; kennethj@niddk.nih.gov (K.A.J.); zhiwei.wen@nih.gov (Z.W.); 3 Institute of Clinical Pharmacology, RWTH Aachen University, Wendlingweg 2, 52074 Aachen, Germany; aparna.malisetty@rwth-aachen.de (A.M.); gschmalzing@ukaachen.de (G.S.)

**Keywords:** P2X5 receptor, voltage clamp, dihydropyridines, purinergic receptor, *Xenopus* oocytes

## Abstract

The P2X5 receptor, an ATP-gated cation channel, is believed to be involved in tumor development, inflammatory bone loss and inflammasome activation after bacterial infection. Therefore, it is a worthwhile pharmacological target to treat the corresponding diseases, especially in minority populations that have a gene variant coding for functional homotrimeric P2X5 channels. Here, we investigated the effects of dihydropyridines on the human full-length P2X5 receptor (hP2X5^FL^) heterologously expressed in *Xenopus* oocytes using the two-microelectrode voltage clamp method. Agonist dependency, kinetics and permeation behavior, including Cl^−^ permeability, were similar to hP2X5^FL^ expressed in HEK293 or 1321N1 cells. Additionally, 1,4-dihydropyridines have been shown to interact with various other purinergic receptors, and we have examined them as potential hP2X5 modulators. Of seven commercially available and four newly synthesized dihydropyridines tested at hP2X5^FL^, only amlodipine exerted an inhibitory effect, but only at a high concentration of 300 µM. Isradipine and—even more—nimodipine stimulated ATP-induced currents in the low micromolar range. We conclude that common dihydropyridines or four new derivatives of amlodipine are not suitable as hP2X5 antagonists, but amlodipine might serve as a lead for future synthesis to increase its affinity. Furthermore, a side effect of nimodipine therapy could be a stimulatory effect on inflammatory processes.

## 1. Introduction

The ATP-activated P2X receptors represent emerging pharmaceutical targets for a variety of conditions [1]. Among the seven P2X receptor subtypes, P2X3 and P2X7 antagonists have already entered clinical trials for chronic cough and inflammatory/neurological diseases, respectively [2,3,4]. Other subtypes have generated extensive structure activity relationship (SAR) studies to discover new and selective agonists and antagonists. For example, P2X4 receptor antagonists are being developed for chronic pain treatment [5]. X-ray and cryo-EM structures of various P2X receptors with agonist or antagonist bounds have been reported [6], which has led to structure-based approaches to understanding the ligand recognition and drug discovery.

Human P2X5 (hP2X5) mRNA is expressed in many tissues, particularly in the brain [7], adipose tissues [8], the immune system [7,9], and various cancer cells [10] (for review, see [11]), including CD34^+^ leukemia progenitor cells [12,13,14]. Despite its unique expression profile and dynamic mRNA regulation, the P2X5 receptor is an outlier in the field of drug development, as no definitive ligand tools have yet been reported [1]. One possible reason is that due to a prevailing T > G SNP (G-allele) at the 3′-splice site of exon 10, most humans express only an apparently non-functional splice variant (see Supplementary Figure S1), designated as hP2X5^Δ328–349^ [7,15,16]. The hP2X5^Δ328–349^ lacks exon 10 encoded residues 328–349, which not only form much of the pore forming second transmembrane domain [16,17] but are also essential for homotrimeric assembly [18]. Only the rare T-allele at the 3′-splice site of exon 10 results in a transcript encoding the 444-residues long, full-length hP2X5 (hP2X5^FL^) subunit, which is capable of assembling into a functional ATP-gated receptor channel [16,17,18]. The rare T-allele has only been detected in tissue samples obtained from African Americans, but not in people of other ethnicities [15].

Another, albeit rare, hP2X5 splice variant that does not translate into a functional hP2X5 receptor channel arises from a single cytosine deletion SNP in exon 3 (rs5818907) [12,13,19]. The induced frameshift results in a different amino acid sequence starting at residue 112, until the elongation stops downstream at position 146 because a stop codon occurs in the new frame. This hP2X5 frameshift variant has been identified as a hematopoietic-restricted minor histocompatibility antigen, LRH-1. Stem cells transplanted from a donor that expresses this frameshift hP2X5 variant induced remission of chronic myeloid leukemia by an LRH-1-specific cytotoxic T cell-mediated lysis in a patient that expressed the wt hP2X5 [12,13,19].

In a recent protein blast search of the National Center for Biotechnology Information (NCBI) database, we failed to find an hP2X5^FL^ sequence () encompassing exon 10. The hP2X5^FL^ DNA used in this work was generated, as in previous work, by genetic insertion of the exon 10 sequence into hP2X5^Δ328–349^ [15,16,18]. We visualized the 3D structure of an hP2X5^FL^ monomer using AlphaFold 2 [20] to localize the exact position of the residues encoded by exon 10 (see Supplementary Figure S2).

The prevailing hP2X5^Δ328–349^ transcript is apparently unique to humans, as it has not been detected in other mammalian species [15]. For example, in rats, only full-length rat P2X5 (rP2X5) mRNA occurs [21,22], which mediates efficient expression of functional rP2X5 homotrimers in cells such as *X. laevis* oocytes [23]. Accordingly, the role of the hP2X5^Δ328–349^ transcript remains enigmatic. Plasma membrane expression of hP2X5^Δ328–349^ protein has been observed in HEK cells [7] and in activated human T lymphocytes [24], but neither in *X. laevis* oocytes [18] nor in 1321N1 cells [15]. Recombinantly expressed hP2X5^Δ328–349^ polypeptide also did not appear at the cell surface when co-expressed with the hP2X5^FL^ subunit or hP2X1. Only coexpression of hP2X5^FL^ with hP2X1 resulted in substantial overlap of the cell surface distribution of both subunits, but the overall pharmacological properties were similar to those observed with individually expressed hP2X1 and hP2X5^FL^ homomers [15].

In this study, we have extended the earlier reports on a technique for characterizing P2X5 receptor activation [16,18]. Furthermore, we focus on the effect of 1,4-dihydropyridines (DHPs) as potential modulators of this receptor. The DHPs initially were found to block L-type Ca^2+^ channels, many of which are used clinically to treat cardiovascular disorders. The DHPs were also noted to be a privileged structure in medicinal chemistry, i.e., they can be sculpted by specific structural changes to bind to unexpected targets, and in some cases with the loss of affinity for L-type Ca^2+^ channels [25,26,27]. Thus, it is conceivable to repurpose this scaffold for diverse targets. In the case of purinergic signaling pathways, various DHPs have been found to inhibit P2X2 receptors, adenosine receptors, and the equilibrative nucleoside transporter (ENT1) [28,29,30].

## 2. Results

### 2.1. Agonist-Dependent Activation of hP2X5^FL^ Receptors

**Figure 1:**
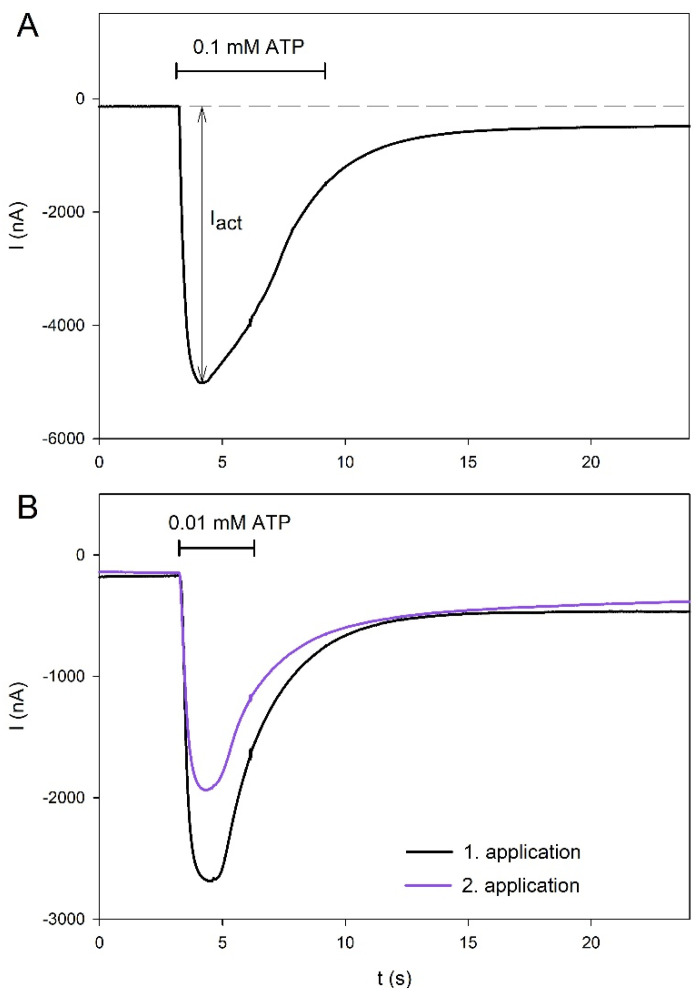
Examples of hP2X5^FL^-mediated current traces in *X. laevis* oocytes elicited by ATP. ATP was applied for 6 s (**A**) or 3 s (**B**) at the indicated ATP concentration and for the duration indicated by the horizontal line. In (**B**), a 2 min washout period was inserted between the first and second ATP applications.

Figure 1 shows examples of ATP-induced currents mediated by the hP2X5^FL^. Application of 0.1 mM ATP for 6 s revealed desensitizing behavior of the hP2X5^FL^ ion channel in the continued presence of ATP (Figure 1A). To keep long lasting desensitization with repeated agonist applications low, as required for the study of the compound effects, we limited the application duration to 3 s and the ATP concentration to 0.01 mM (Figure 1B). Under these conditions, the mean amplitude of the 2nd ATP-induced current was 72 ± 7% (N = 10 oocytes) of the amplitude of the preceding application of 0.01 mM ATP. In all further experiments, the current amplitudes were related accordingly as

(1) $$I_{\mathrm{act},\mathrm{rel},0.01\mathrm{ATP}}=\frac{I_{\mathrm{act}}}{I_{\mathrm{act},0.01\mathrm{ATPbefore}}}$$

with I_act_ measured as the maximum ATP-induced inward current as described in Figure 1A, and I_act,rel,0.01ATP_ being I_act_ measured during application of 0.01 mM ATP 3 min before.

**Figure 2:**
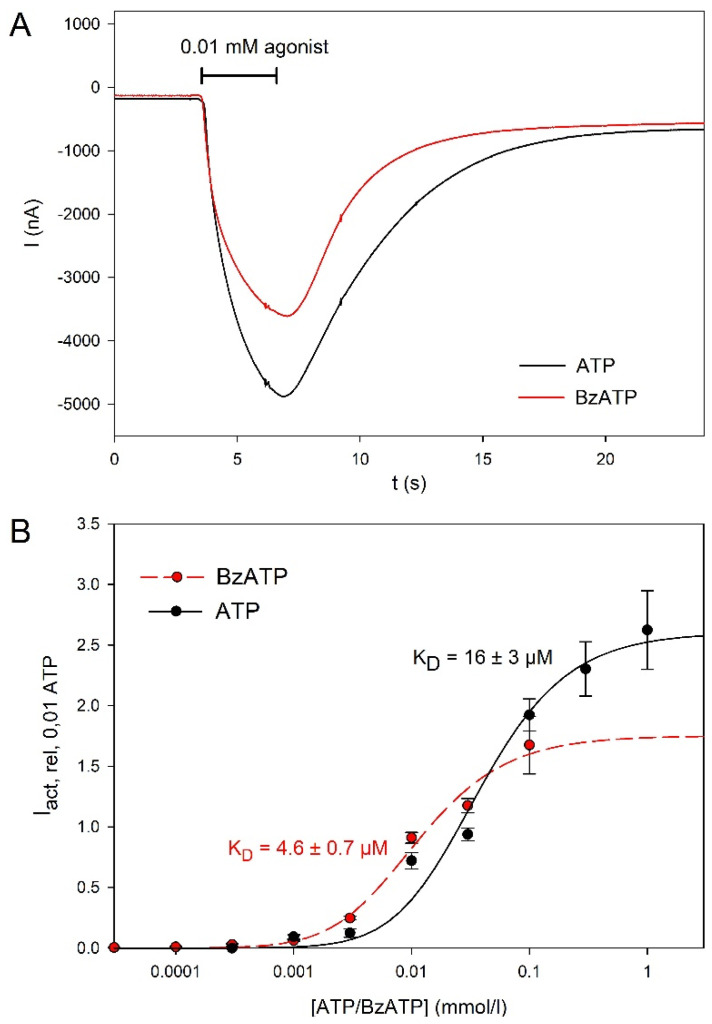
Agonist dependence of hP2X5^FL^-mediated ion currents in *X. leavis* oocytes. (**A**) Examples of current traces elicited consecutively by ATP or BzATP as indicated in the same hP2X5^FL^-expressing oocyte. (**B**) Concentration dependence of currents evoked by the indicated agonist. I_act,rel,0.01ATP_ was calculated according to Equation (1) and the approximation was performed using Equation (2). Data are means ± SEM from of 4–12 oocytes.

The dependence of hP2X5^FL^-mediated currents on the concentrations of the two agonists, ATP and BzATP, is shown in Figure 2. The best approximation was achieved with the following modified Hill function [31]:

(2) $$I_{\mathrm{act},\mathrm{rel},0.01\mathrm{ATP}}\left(\left[\mathrm{agonist}\right]\right)=\frac{I_{\mathrm{act},\max}}{{\left(1+\frac{K_{D}}{\left[\mathrm{agonist}\right]}\right)}^{2}}$$

where K_D_ is the apparent agonist dissociation constant of the activation sites and I_act_,_max_ is the maximal relative amplitude at agonist concentrations saturating the activation sites. The most potent P2X7R agonist, BzATP, displayed a higher affinity for hP2X5^FL^ than ATP but a lower efficacy, as the maximum relative amplitudes were 2.60 ± 0.13 for ATP and 1.75 ± 0.08 for BzATP (*p* < 0.05).

### 2.2. Permeation Behavior of hP2X5^FL^ Receptor Ion Channels

**Figure 3:**
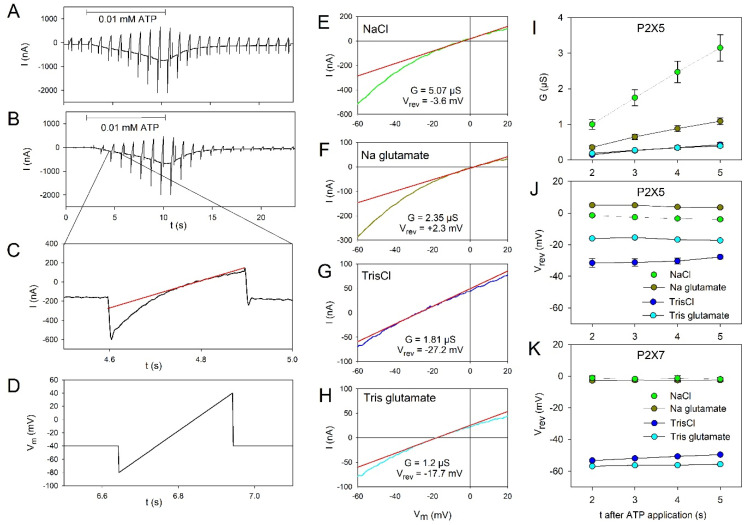
Permeation behavior of oocyte-expressed hP2X5^FL^ ion channels. Time course of ramp currents during voltage ramps applied every second before and during application of 0.01 mM ATP for the time indicated by the horizontal bar before (**A**) and after (**B**) subtraction of the resting ion currents. The bathing solution was a NaCl-based Ringer’s solution. (**C**) Current ramp from a section of B as indicated. The red straight line is the linear fit of the current around the reversal potential. (**D**) Voltage ramp protocol. (**E**–**H**) Examples of ramp currents induced by 0.01 mM ATP in bathing solutions with the indicated main extracellular ions. The ramp current recorded before the ATP application was subtracted. The red lines indicate the linear approximation of the current–voltage dependence near I = 0, from which the slope conductance G and the reversal potential V_rev_ are obtained as indicated. Dependence of G (**I**) and V_rev_ (**J**,**K**) on the extracellular ions of hP2X5^FL^ (**I**,**J**) and hP2X7 (**K**) expressing oocytes. All mean values are significantly different except the reversal potentials in NaCl and Na glutamate solution for hP2X7 expressing oocytes. Means from 13–37 oocytes.

The permeation behavior was investigated by applying voltage ramps during ATP-induced hP2X5^FL^ receptor activation [32,33]. The principle of the measurements is shown in Figure 3A–D. Examples of the determination of the reversal potential of the voltage ramp-induced currents are shown for hP2X5^FL^-expressing oocytes in bathing solutions containing NaCl (Figure 3E), Na glutamate (Figure 3F), TrisCl (Figure 3G) and Tris glutamate as the main ions (Figure 3H). The statistical summary is shown in Figure 3I–K. The hP2X5^FL^-conductance was greatly reduced when Cl^−^ was replaced by the larger anion glutamate^−^ and even more so when Na^+^ was also replaced by the larger cation Tris^+^ (Figure 3I). The dependence of the reversal potential on the cation and anion species on the extracellular solution (Figure 3J) shows that the hP2X5^FL^ ion channel pore is permeable to both anions and cations.

For comparison, the same experiments were performed with hP2X7 receptor-expressing oocytes (Figure 3K). The negative shift of the reversal potential after replacement of Na^+^ by Tris^+^ and the absence of a positive shift of V_rev_ after replacement of Cl^−^ by glutamate^−^ confirm the exclusive cation selectivity of the hP2X7 receptor [32,34,35] under identical experimental conditions.

### 2.3. Voltage Dependence of hP2X5^FL^ Receptor Ion Channels

**Figure 4:**
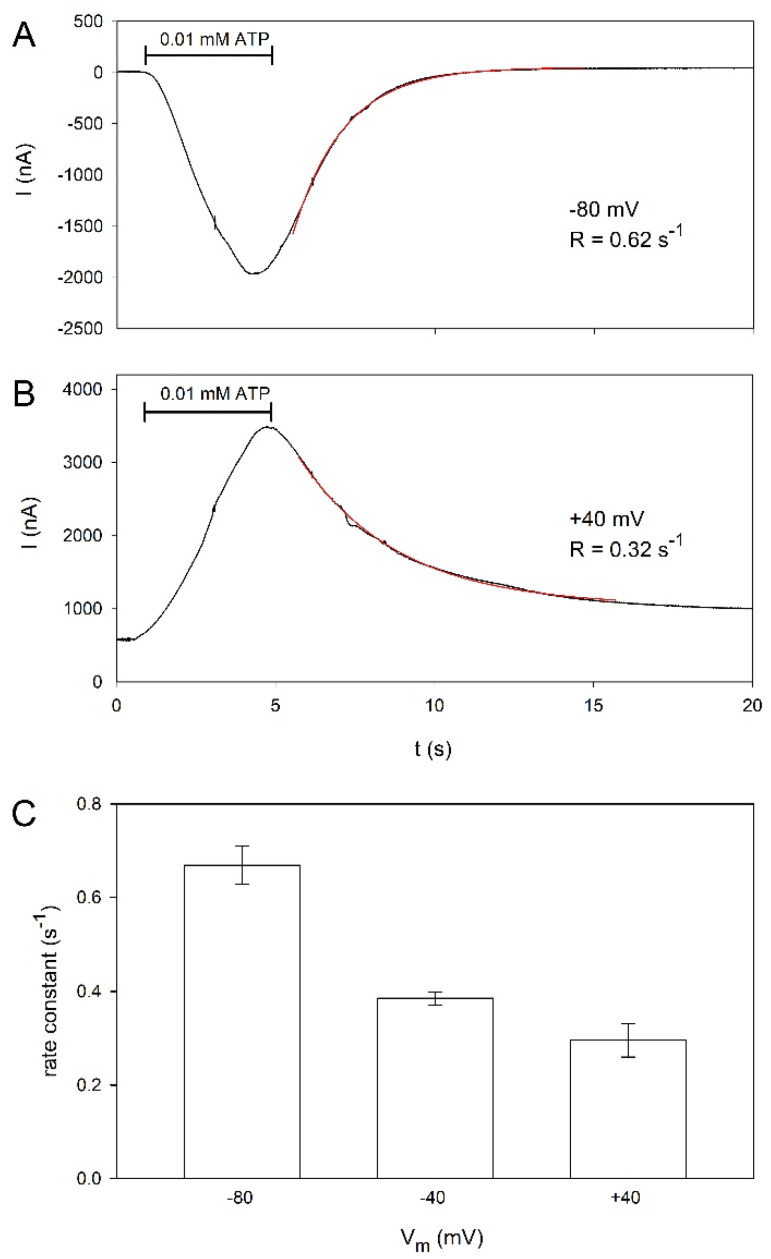
Voltage dependence of oocyte-expressed hP2X5^FL^ ion channels. Characteristic example of hP2X5^FL^-dependent current evoked by 0.01 mM ATP for the time indicated by the horizontal bar at a membrane potential of −80 mV (**A**) or +40 mV (**B**), respectively. The exponential fits of the deactivation time courses according to eq. 3 are plotted as red lines, with the determined rate constants as indicated. (**C**) Statistics of the effect of the membrane potential on the deactivation rate constant. All mean values are significantly different from each other. Means are from 9–19 oocytes.

Because voltage-dependent kinetics have been described for hP2X5^FL^ receptors heterologously expressed in HEK cells [16], we tested whether the deactivation of hP2X5^FL^ receptors in oocytes is similarly dependent on the membrane potential. Figure 4A,B shows examples of ATP-induced currents measured at membrane voltages of −80 and +40 mV. The deactivation of the currents during ATP washout was approximated by

(3) $$I_{\mathrm{deact}}\left(t\right)=I_{\max}e^{-\mathrm{Rt}}$$

where I_max_ is the maximum amplitude of the deactivating current and R is the rate constant. The deactivation slows down at more positive membrane potentials, as shown in Figure 4C.

### 2.4. Effect of Dihydropyridines on hP2X5^FL^-Mediated Currents

**Figure 5:**
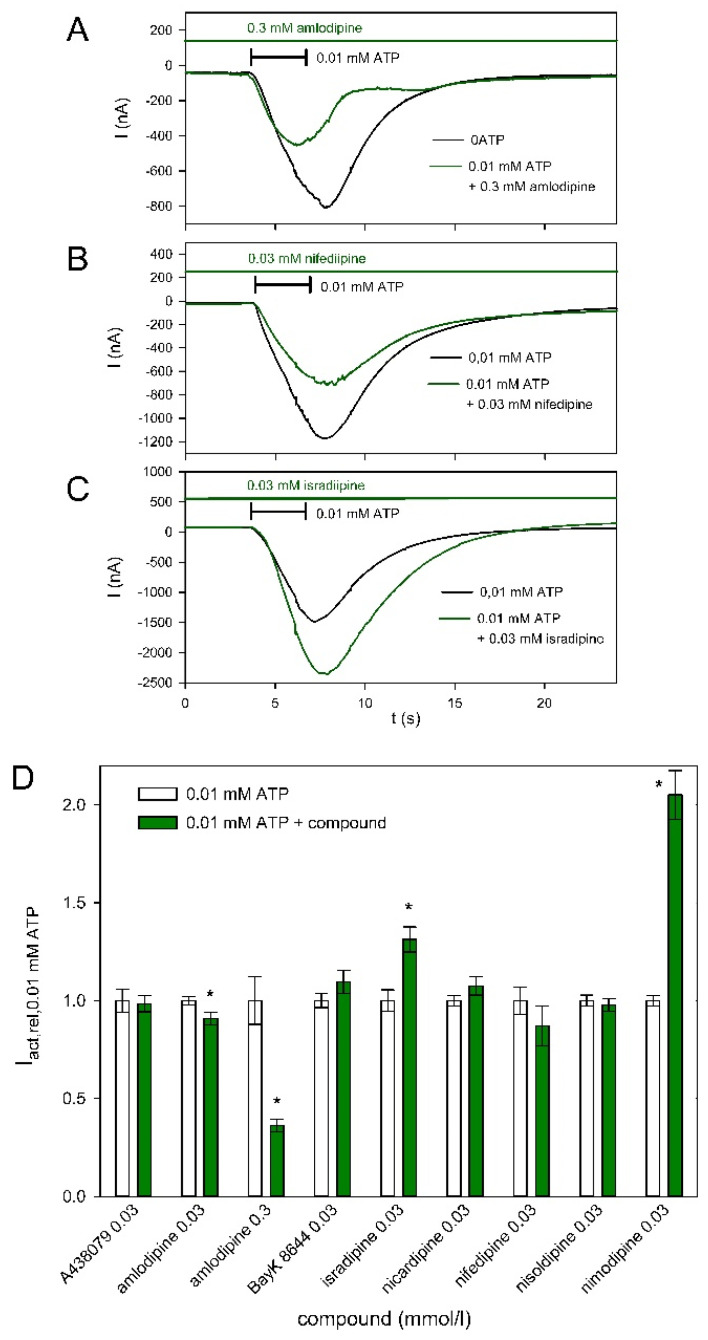
Effect of different dihydropyridines and the P2X7-specific blocker A438079 on hP2X5^FL^-mediated currents. Examples of the inhibitory effect of amlodipine (**A**) the effect of nifedipine (**B**) and the stimulatory effect of isradipine (**C**) on ATP-induced currents. Currents were evoked by ATP in bathing solution and 2 min later in bathing solution supplemented with the indicated dihydropyridine derivative. (**D**) Summary of the effects of A438079 and various dihydropyridines on ATP-induced currents. Means are normalized to the mean value of the currents measured during the second ATP application without additional substances (control). Means of 9–50 oocytes. Values that differ significantly from the ATP-only control are marked by asterisks.

Next, we tested the effect of various compounds, i.e., the P2X7-specific blocker A438079 and several dihydropyridines such as amlodipine on ATP-induced hP2X5^FL^-dependent currents. Examples for the effect of amlodipine (Figure 5A) and isradipine (Figure 5B) demonstrate inhibitory and stimulatory effects of dihydropyridines on hP2X5^FL^-mediated currents. Figure 5C summarizes the effects of the tested compounds. At 30 µM, only amlodipine exerted a small inhibitory effect. In contrast, isradipine and nimodipine stimulated the ATP-induced currents.

**Figure 6:**
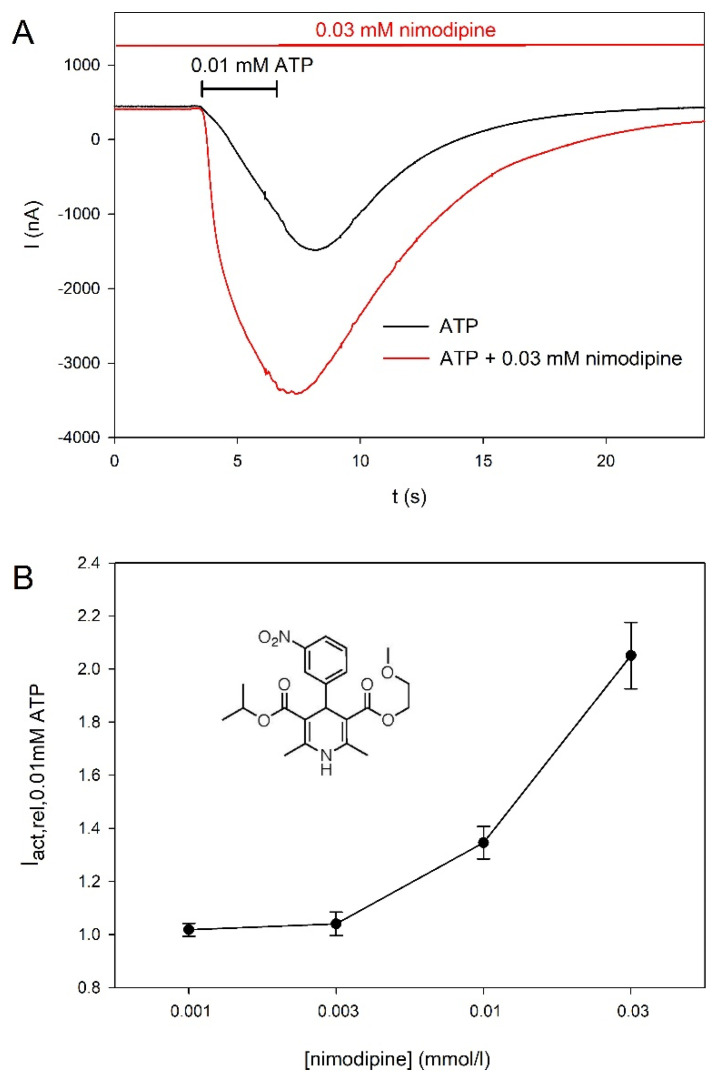
Concentration-dependent effect of nimodipine on hP2X5^FL^-mediated currents. (**A**) Example current traces consecutively evoked by ATP and ATP + nimodipine. (**B**) Concentration dependence of the effect of nimodipine on ATP-elicited hP2X5^FL^-mediated currents. Means are from of 6–40 oocytes. The structure of nimodipine is shown in the inset.

The strong stimulatory effect of nimodipine was then investigated in more detail. Figure 6A demonstrates an example of the nimodipine effect. In Figure 6B, the concentration dependence of the stimulatory effect of nimodipine is shown. The stimulatory effect of nimodipine is already obvious at 10 µM. We could not measure a saturating nimodipine concentration since nimodipine was not soluble at concentrations higher than 30 µM.

**Figure 7:**
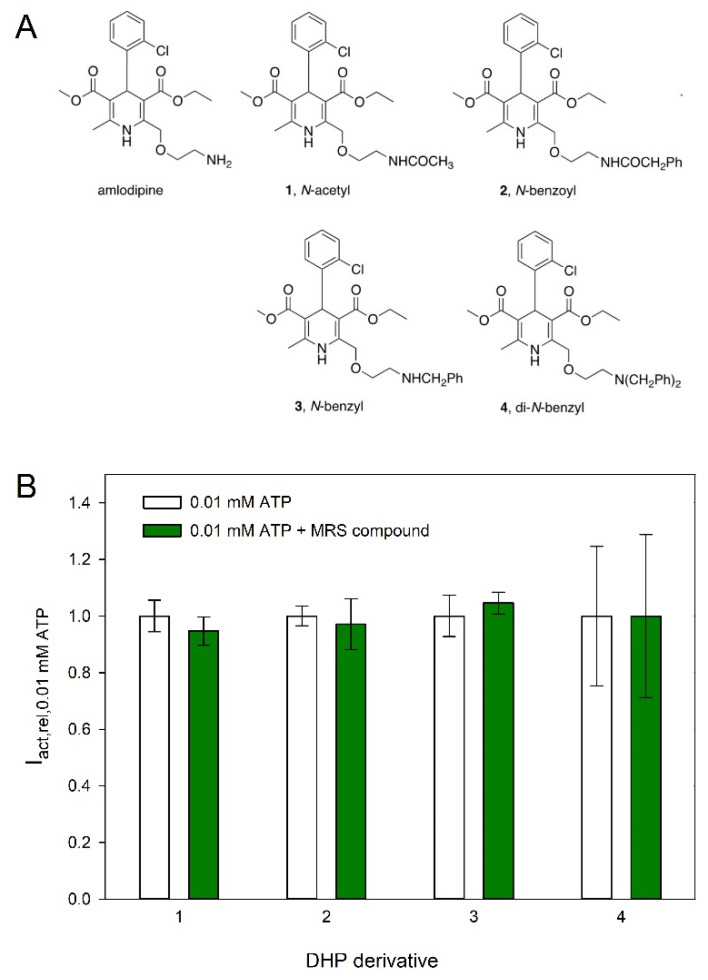
Effects of amlodipine-derived compounds on hP2X5^FL^-mediated currents. (**A**) Structures of amlodipine and four newly synthesized DHPs derived from amlodipine. (**B**) Statistics of the effect of the MRS compounds shown in A. Means are from of 7–9 oocytes.

**Figure 8:**
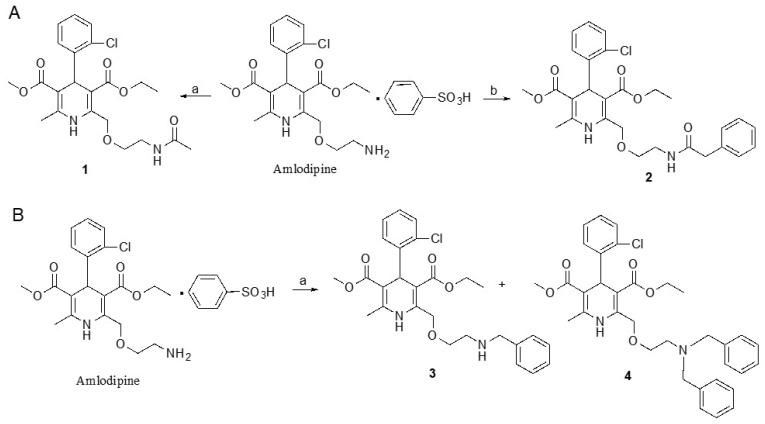
Synthesis of new dihydropyridine derivatives. (**A**) Preparation of amide derivatives **1** and **2** of amlodipine. Reagents and conditions: (a) acetyl chloride, pyridine, 0 °C—rt., overnight, 44%; (b) phenylacetyl chloride, TEA, CH_2_Cl_2_, 0 °C—rt, overnight, 93%. (**B**) Preparation of *N*-benzylated derivatives of amlodipine. Reagents and conditions: (a) benzyl bromide, K_2_CO_3_, reflux, 3.5 h, 37% **3** and 39% **4**.

Based on the small inhibition of hP2X5^FL^-mediated currents by amlodipine, four additional congeners of this DHP were synthesized (Figure 7 and Figure 8). The site of derivatization was the primary ethylamino side chain of amlodipine, which is relatively easily modified through acylation or alkylation reactions. Amidation of lead amlodipine with acetyl chloride and phenylacetyl chloride in the presence of bases offered amide derivatives 1 and 2, respectively (Figure 8A). *N*-Benzylation of amlodipine with one equivalent of benzyl bromide led to the formation of mono-benzylated derivative 3 in addition to di-benzylated derivative 4 (Figure 8B). However, the activity of amlodipine was not maintained in these derivatives (Figure 7).

## 3. Discussion

### 3.1. Activation and Kinetics of hP2X5^FL^-Mediated Currents

The ATP concentration dependence of the hP2X5^FL^-dependent currents differs from those measured in HEK293 cells [16] in several ways. The EC_50_ measured in oocytes was ≈39 µM, which is about 10 times higher than the value measured in HEK cells. This difference is surprising because the HEK cell experiments were performed in extracellular solutions containing Ca^2+^ and Mg^2+^, which are known to chelate ATP^4−^. Indeed, ATP^4−^ is the true agonist at most P2X receptors, including P2X2, P2X4 [36] and P2X7 [37]. If ATP^4−^ is also the agonist at hP2X5^FL^, we would expect a leftward shift of the concentration-response curve in the divalent free extracellular solutions used for the oocyte experiments. Although we were able to fit the [ATP^4−^] dependency with a single Hill function, a closer look at Figure 2 suggests a biphasic [ATP^4−^] dependence of the current amplitudes, similar to hP2X7 receptors [37].

The kinetics of the chicken P2X5 receptor is strongly dependent on the extracellular Ca^2+^ concentration [38]. Therefore, the Ca^2+^ dependence of hP2X5^FL^ should be investigated in more detail in the future. The determination of the concentration dependence of BzATP is also somehow uncertain, as a clear saturating concentration was not reached. However, we can conclude that at least at low concentrations, there is not much difference between the effects of ATP and BzATP, in contrast to hP2X4, where BzATP is less potent, and hP2X7, where BzATP is more effective and potent than ATP [39]. Earlier efforts to characterize ligand interactions with the P2X5^FL^ [15] determined EC50 values for agonists (ATP, 0.3 µM; α,β-me-ATP, 12.2 µM) and IC50 values for several known nonselective P2R antagonists (PPADS 0.65 µM; suramin 13 µM; TNP-ATP 2.5 µM).

Similar to P2X5^FL^ expressed in HEK cells [16], the deactivation measured in oocytes becomes faster with more positive potentials, although deactivation in oocytes at +40 mV is significantly slower than in HEK cells. The voltage dependence cannot yet be explained based on the P2X5 structure because P2X receptors have no known voltage-sensitive domain, and the ATP binding site is not located within the electrical field of the membrane.

### 3.2. Permeation Characteristics of hP2X5^FL^

A peculiar characteristic of hP2X5^FL^ is its chloride permeability, which was previously found for the chicken P2X5 receptor [16,38]. With a slight modification of the experimental setup, i.e., the used extracellular solutions for determination of the hP2X5^FL^ permeability, we confirmed the dependence of the hP2X5^FL^-mediated currents on the extracellular Cl^−^ concentration, indicating that the hP2X5^FL^ conducts Cl^−^ ions. The permeability for cations and anions is reflected by the shift of the reversal potential and the decrease of the conductance when Na^+^ or Cl^−^ were replaced by the respective larger ions. Although Tris^+^ and N-methyl-d-glucamine (NMDG^+^) are protonated amines of similar size (MW < 200) [35], the shift of the reversal potential by substitution of Na^+^ by Tris^+^ was considerably higher than after substitution by NMDG^+^ for unknown reasons [16]. The structural basis for the anion permeability of hP2X5^FL^ remains unresolved, as there are only minor structural differences compared to the exclusively cation-permeable P2X2, P2X2/3 [16], and human P2X7 receptors [32,35].

### 3.3. Effect of Dihydropyridines on hP2X5^FL^

Specific blockers of P2X5 receptors are unknown to date [1]. However, it was reported recently that α,β-methylene ATP-induced contractions were inhibited by nifedipine in rat vascular smooth muscle cells [40] and that nicardipine reduced the current induced at recombinant rat P2X2 and P2X4 receptors expressed in *Xenopus* oocytes [28]. Therefore, we investigated the effect of several dihydropyridines on hP2X5^FL^. Most of the tested compounds were without effect. Only amlodipine exerted a weak inhibitory effect at 30 µM. In addition, several amlodipine derivatives were without effect, such as the weak P2X2 antagonist nicardipine. Surprisingly, we observed stimulatory effects of nicardipine and—even stronger—of nimodipine on hP2X5^FL^-mediated currents. The enhancement of hP2X5^FL^-mediated currents by certain dihydropyridines is likely allosteric, as it requires both the agonist and modulator to be present. Both positive and negative allosteric modulators of other P2X receptor subtypes have already been reported and in some cases characterized structurally [41,42].

In rodents, activation of P2X5 receptors mediates differentiation of skeletal muscle satellite cells [43], senses ischemia in skeletal muscles [44], and mediates cell differentiation and has in this way antineoplastic effects [45]. Therefore, the results presented here may help to find specific pharmacological tools to target P2X5 receptors to determine the influence on P2X5-mediated physiological and pathophysiological effects. Furthermore, the possibility of side effects of the clinically used dihydropyridines due to P2X5 receptor modulation, such as a stimulatory effect on inflammatory processes, has to be kept in mind. The relevance to humans of the effect of P2X5-mediated bone loss in mice [45,46,47] remains to be explored. Since the T allele of P2X5 that produces the full-length functional receptor occurs predominantly in people of African background, the relevance of P2X5 antagonists to minority health issues such as diabetes should be explored [8,48]. It is estimated that the T allele is present in only 14% of the general population [16] but in 70% of African Americans [15].

The inactivity of four new derivatives of weak P2X5 antagonist amlodipine suggested that its primary aliphatic amino position is bound within a restrictive portion of the DHP binding site on the receptor. Nevertheless, amlodipine might serve as a lead for future synthesis at positions other than the amino group to increase its P2X5 receptor affinity. It would be useful to study how these DHPs affect P2X5 receptor heterotrimers [15,49] in addition to the homotrimer studied here. Additionally, other known dihydropyridine drugs may serve as chemical leads for more specific pharmacological agents targeting the human P2X5 receptor.

## 4. Materials and Methods

### 4.1. Reagents

Unless otherwise stated, chemicals and molecular biology reagents were purchased from Sigma (Sigma-Aldrich, Taufkirchen, Germany), Merck (Darmstadt, Germany), Tocris (Wiesbaden, Germany), and New England Biolabs (Schwalbach, Germany). Na_2_ATP was purchased from Roche (Mannheim, Germany) and 3’-O-(4-benzoyl)benzoyl-ATP (BzATP) from Sigma.

The dihydropyridines (DHPs) were purchased from Tocris (Bristol, UK). They were solubilized in DMSO as stock 1 mM solutions and then diluted in the bathing solution (see below) to the given concentration.

### 4.2. Construction of Full-Length hP2X5 mRNA

We subcloned the exon 10 missing hP2X5 cDNA (hP2X5^Δ^^328–349^) into our expression vector pNKS2 [50] by PCR from a human brain cDNA library (Invitrogen) as detailed previously [18]. To generate the full-length hP2X5^FL^, we executed several consecutive rounds of QuickChange mutagenesis [51] to insert the 22 codons for human exon 10 into hP2X5^Δ^^328–349^. Our final hP2X5^FL^ encoding construct [18] deviates in two residues, L216F and F236L, from the construct described by Le et al. [7] and Bo et al. [16]. Finally, we inserted the sequence for an N-terminal hexahistidine tag by QuickChange mutagenesis to yield His-hP2X5^FL^, which we used in this work. We synthesized capped cRNA from the XhoI-linearized His-hP2X5^FL^ plasmid using the described methods [52,53].

### 4.3. Expression and of hP2X5^FL^ in Oocytes of Xenopus Laevis

The surgically removed *X. laevis* oocytes were defolliculated by treatment with collagenase (NB 4G, Serva, Heidelberg, Germany). Oocytes at Dumont stages V–VI were injected with 23 or 46 nl of cRNAs diluted to 0.1 to 1 µg/µL for hP2X5^FL^ or 0.05 µg/µL for hP2X7, respectively, to keep the maximal ATP-elicited current amplitudes < 5 μA to limit the current-induced stress for the oocytes. The oocytes were incubated at 19 °C in a sterilized frog Ringer’s solution (Mg/Ca-ORi: 100 mM NaCl, 2.5 mM KCl, 1 mM MgCl_2_, 1 mM CaCl_2_, and 10 mM HEPES, pH 7.4) and supplemented with penicillin (100 U/mL) and streptomycin (100 μg/mL).

### 4.4. Voltage Clamp Measurements

Two microelectrode measurements and data evaluation were performed as already described [32,33] 1–3 days after cRNA injection. Microelectrodes filled with 3 M KCl with resistances of 0.8–1.2 MΩ were impaled into oocytes superfused with a frog Ringer’s solution (ORi: 90 mM NaCl, 2.5 mM KCl, 1 mM CaCl_2_, 1 mM MgCl_2_, 10 mM HEPES). Currents were recorded at a holding potential of −40 mV at room temperature (~22 °C) using an oocyte clamp OC-725C amplifier (Warner Instruments, Hamden, USA), filtered at 100 Hz and sampled at 85 Hz. Switching between the different bathing solutions was achieved within less than 1 s by a set of computer-controlled magnetic valves using a modified U-tube technique. Measurements of the hP2X5R^FL^-dependent currents were performed in a bathing solution consisting of 100 mM NaCl, 2.5 mM KCl, 0.1 mM flufenamic acid, and 5 mM HEPES. Ca^2+^ and Mg^2+^ were omitted to avoid the activation of endogenous currents by Ca^2+^ influx through the P2X7R channels and to prevent the complexation of ATP^4−^. The used concentrations of ATP^4−^ are therefore denoted as [ATP]. Flufenamic acid was added to the Ca^2+^-free solutions in order to block the conductance evoked by the omission of external divalent cations. The hP2X5R^FL^-mediated inward currents were elicited by switching for 3 or 6 s to a bathing solution that also contained ATP or BzATP at the concentrations indicated in the text and figures. The interval between agonist applications was usually 3 min.

Ramp currents were measured during 300 ms long voltage ramps, going from −80 to +40 mV, applied every 1 s. The holding potential between the ramps was maintained at −40 mV. The ATP-induced ramp currents were calculated as the difference between the ramp currents before and during the ATP application. For measuring the dependence of the reversal potential of the ramp currents on cations or anions, we omitted K^+^ ions and reduced the remaining extracellular Ca^2+^ concentration of the nominally Ca^2+^ free bathing solution by adding 0.1 mM EGTA (NaClRi: 100 mM NaCl, 0.1 mM flufenamic acid, 5 mM HEPES, 0.1 mM EGTA). We also replaced small ions by larger ones, namely Na^+^ by Tris^+^ (TrisClRi: 100 mM TrisCl, 0.1 mM flufenamic acid, 0.1 mM EGTA), Cl^−^ by glutamate^−^ (Na glutamateRi: 100 mM Na glutamate, 0.1 mM flufenamic acid, 5 mM HEPES, 0.1 mM EGTA), or both Na^+^ and Cl^−^ by Tris^+^ glutamate^−^ (Tris glutamateRi: 100 mM Tris glutamate, 0.1 mM flufenamic acid, 0.1 mM EGTA). The pH of all solutions was adjusted with NaOH to 7.4.

### 4.5. Data Analysis and Presentation

The data were stored and analyzed on a personal computer using software programmed in our department (Superpatch 2000, SP-Analyzer by T. Böhm). For approximations, statistical analysis, and presentation of the data, we used the SigmaPlot software (Systat Software). Statistical data were given as means ± SEM and analyzed using one-way ANOVA. We tested the statistical significance of the differences between means using the Bonferroni’s t-test for multiple comparisons. Statistical significance was set at a *p*-value of <0.05.

### 4.6. Chemical Synthesis

Reagents and instrumentation: All chemicals and anhydrous solvents were obtained directly from Sigma-Aldrich (St. Louis, MO, USA) or other commercial sources. All reactions were carried out under an argon atmosphere using anhydrous solvents. Room temperature (rt) refers to 25 ± 5 °C. Column chromatography utilized silica gel (40–63 μm, 60 Å). A 400 MHz NMR spectrometer (Bruker Scientific Instruments, Billerica, MA, USA) and high-resolution mass spectrometry (HRMS), performed on a proteomics optimized Micromass Q-TOF-2 (Waters Corp., Milford, MA, USA), were used to confirm the structural identity of the synthesized compounds. Chemical shifts for ^1^H-NMR spectra are given in ppm (δ), calibrated to the residual solvent signal peak of DMSO-*d*_6_ (2.50 ppm) for ^1^H NMR with coupling constant (*J*) values reported in Hz.

2-[[2-(*N*-Acetylamino)ethoxy]methyl]-4-(2-chlorophenyl)-3-(ethoxycarbonyl)-5-(methoxycarbonyl)-6-methyl-l,4-dihydropyridine (**1**).

Acetyl chloride (3.2 μL, 3.5 mg, 0.045 mmol) was added dropwise to a stirred, ice-cooled solution of amlodipine *p*-toluenesulfonate (17.0 g, 0.03 mmol) in pyridine (0.5 mL). The mixture was stirred at rt overnight. The volatiles were evaporated and the residue was column chromatographed (CH_2_Cl_2_/MeOH, 100:0 → 95:5) and further purified on RP-HPLC (*t*_R_ = 40 min; C18; A: ACN, B: H_2_O; 0% A → 100% A in 40 min, flow rate = 5 mL/min) to give **2** as a white solid (6.0 mg, 44%): ^1^H NMR (400 MHz, DMSO) δ 8.46 (s, 1H), 7.99 (t, *J* = 5.3 Hz, 1H), 7.31 (d, *J* = 7.7 Hz, 1H), 7.28–7.19 (m, 2H), 7.12 (t, *J* = 7.5 Hz, 1H), 5.29 (s, 1H), 4.62 (d, *J* = 14.2 Hz, 1H), 4.53 (d, *J* = 14.1 Hz, 1H), 4.03–3.90 (m, 2H), 3.50 (s, 3H), 3.45 (d, *J* = 5.1 Hz, 2H), 3.28–3.20 (m, 2H), 2.30 (s, 3H), 1.83 (s, 3H), 1.10 (t, *J* = 7.1 Hz, 3H). HRMS *m*/*z* [M + H]^+^ for C_22_H_27_ClN_2_O_6_ calculated 451.1636, found 451.1632.

2-[[2-(*N*-Phenylacetylamino)ethoxy]methyl]-4-(2-chlorophenyl)-3-(ethoxycarbonyl)-5-(methoxycarbonyl)-6-methyl-l,4-dihydropyridine (**2**).

Phenylacetyl chloride (3.6 μL, 4.2 mg, 0.027 mmol) was added dropwise to a stirred, ice-cooled solution of amlodipine *p*-toluenesulfonate (10.2 mg, 0.018 mmol) and TEA (7.5 μL, 5.5 mg, 0.054 mmol) in CH_2_Cl_2_ (0.5 mL). The mixture was stirred at rt overnight. The volatiles were evaporated and the residue was column chromatographed (CH_2_Cl_2_/MeOH, 100:0 → 95:5, with 1% TEA) and further purified on preparative TLC (CH_2_Cl_2_/MeOH = 97:3, with 1% TEA) to give **3** as a white solid (8.8 mg, 93%): ^1^H NMR (400 MHz, DMSO) δ 8.40 (s, 1H), 8.20 (t, *J* = 5.3 Hz, 1H), 7.31–7.14 (m, 8H), 7.09 (t, *J* = 7.5 Hz, 1H), 5.26 (s, 1H), 4.59 (d, *J* = 14.1 Hz, 1H), 4.50 (d, *J* = 14.2 Hz, 1H), 4.00–3.88 (m, 2H), 3.47 (s, 5H), 3.41 (s, 2H), 3.27 (d, *J* = 5.1 Hz, 2H), 2.21 (s, 3H), 1.07 (t, *J* = 7.1 Hz, 3H). HRMS *m*/*z* [M + H]^+^ for C_28_H_31_ClN_2_O_6_ calculated 527.1949, found 527.1946.

2-[[2-(*N*-Benzylamino)ethoxy]methyl]-4-(2-chlorophenyl)-3-(ethoxycarbonyl)-5-(methoxycarbonyl)-6-methyl-l,4-dihydropyridine (**3**) and 2-[[2-(*N,N*-dibenzylamino)ethoxy]methyl]-4-(2-chlorophenyl)-3-(ethoxycarbonyl)-5-(methoxycarbonyl)-6-methyl-l,4-dihydropyridine (**4**).

A mixture of amlodipine *p*-toluenesulfonate (22.7 mg, 0.04 mmol), benzyl bromide (4.8 μL, 6.8 mg, 0.04 mmol), and K_2_CO_3_ (11 g, 0.08 mmol) in CH_3_CN (1.5 mL) was heated under reflux for 3.5 h. The volatiles were evaporated, and the residue was column chromatographed (CH_2_Cl_2_/MeOH, 100:0 → 95:5, with 1% TEA). Appropriate fractions were combined and evaporated to give **4** as a white solid (9.1 mg, 39%): ^1^H NMR (400 MHz, DMSO) δ 8.53 (s, 1H), 7.39–7.19 (m, 11H), 7.16 (t, *J* = 7.3 Hz, 1H), 7.10 (t, *J* = 7.5 Hz, 1H), 5.29 (s, 1H), 4.55 (d, *J* = 13.7 Hz, 1H), 4.49 (d, *J* = 13.7 Hz, 1H), 4.00–3.88 (m, 2H), 3.58 (d, *J* = 14.5 Hz, 6H), 3.49 (s, 3H), 2.62 (t, *J* = 5.8 Hz, 2H), 2.22 (s, 3H), 1.08 (t, *J* = 7.1 Hz, 3H). HRMS *m*/*z* [M + H]^+^ for C_34_H_37_ClN_2_O_5_ calculated 589.2469, found 589.2473. Appropriate fractions of **3** were collected and further purified on preparative TLC (CH_2_Cl_2_/MeOH, 97:3, with 1% TEA) to give **3** as a white solid (7.3 mg, 37%): ^1^H NMR (400 MHz, DMSO) δ 8.89 (s, 1H), 7.42–7.19 (m, 7H), 7.12 (t, *J* = 7.5 Hz, 1H), 5.29 (s, 1H), 4.65 (d, *J* = 14.9 Hz, 1H), 4.54 (d, *J* = 14.9 Hz, 1H), 4.00–3.89 (m, 2H), 3.75 (s, 2H), 3.55 (t, *J* = 4.7 Hz, 2H), 3.49 (s, 3H), 2.71 (d, *J* = 6.3 Hz, 2H), 2.21 (s, 3H), 1.09 (t, *J* = 7.0 Hz, 3H). HRMS *m*/*z* [M + H]^+^ for C_27_H_31_ClN_2_O_5_ calculated 499.2000, found 499.1995.

## Data Availability

Data supporting the reported results can be found at https://opendata.uni-halle.de//handle/1981185920/66415 (accessed on 10 January 2022).

## Supplementary Materials

The following supporting information can be downloaded at: https://www.mdpi.com/article/10.3390/molecules27061846/s1, Figure S1—Amino acid sequence of the hP2X5^FL^ subunit compared with best hits in the NCBI protein database. The complete protein sequence of the hP2X5^FL^ clone used in the present study was aligned with the NCBI database using the BLAST webpage. Available online: https://blast.ncbi.nlm.nih.gov/Blast.cgi?PAGE=Proteins (accessed on 10 January 2022). The best hit is the exon 10-deleted sequence ID NM_002561 (equal to UniProt Q93086.4) [7,16]. Our hP2X5^FL^ sequence differs from NM_002561/Q93086.4 by having one leucine residue each at positions 216 and 232 (highlighted in green) instead of a phenylalanine. The mutagenically inserted 22 codons of human exon 10 (GenBank ID AF168787 [16]) are highlighted in yellow-green; within this 22 residues, the 3 residues in bold italics are conservatively different in rat P2X5. The bolt-blue underlined stretches of residues mark the transmembrane domains TM1 and TM2. The residue stretches highlighted in red and salmon refer to the head domain and the left flipper, which are shown in Supplementary Figure S2 with the same color code. Also shown are individual residues involved in ATP binding in lime-green. Figure S2—3D structure of a hP2X5^FL^ protomer modelled with AlphaFold 2. The 3D structure of the hP2X5^FL^ subunit was modelled by using AlphaFold 2 (https://cryonet.ai/af2/, accessed on 10 January 2022) and visualized using Pymol version 2.5.2 (http://www.pymol.org/, accessed on 10 January 2022). Residues encoded by exon 10 are highlighted in light green and include the pre-TM2 region and the following two-thirds of TM2. For better orientation, the head domain and the left flipper are highlighted in red and salmon, respectively. The two leucine residues, which are phenylalanines in the NCBI sequence NM_002561 [16], are indicated in magenta in the stick presentation. In addition, conserved residues involved in ATP binding as green sticks.

## Sample Availability

Samples of the compounds are not available.
