# Supplementary material for: Dihydropyridines Potentiate ATP-Induced Currents Mediated by the Full-Length Human P2X5 Receptor
Source: Molecules. 2022 Mar 11;27(6):1846. doi: 10.3390/molecules27061846 (PMC8948676; doi:10.3390/molecules27061846)
Supplement: Supplementary file 1 [file molecules-27-01846-s001.zip › molecules-1618041-supplementary.pdf]

Supplementary Materials:

Supplementary Figure S1—Amino acid sequence of the hP2X5^{FL} subunit compared with best hits in the NCBI protein database. The complete protein sequence of the hP2X5^{FL} clone used in the present study was aligned with the NCBI database using the BLAST webpage. Available online: https://blast.ncbi.nlm.nih.gov/Blast.cgi?PAGE=Proteins (accessed on 10 January, 2022). The best hit is the exon 10-deleted sequence ID NM_002561 (equal to UniProt Q93086.4) [1,2]. Our hP2X5^{FL} sequence differs from NM_002561/Q93086.4 by having one leucine residue each at positions 216 and 232 (highlighted in green) instead of a phenylalanine. The mutagenically inserted 22 codons of human exon 10 (GenBank ID AF168787 [2]) are highlighted in yellow-green; within this 22 residues, the 3 residues in bold italics are conservatively different in rat P2X5. The bolt-blue underlined stretches of residues mark the transmembrane domains TM1 and TM2. The residue stretches highlighted in red and salmon refer to the head domain and the left flipper, which are shown in Supplementary Figure S2 with the same color code. Also shown are individual residues involved in ATP binding in lime-green.

Supplementary Figure S2—3D structure of a hP2X5^{FL} protomer modelled with AlphaFold 2. The 3D structure of the hP2X5^{FL} subunit was modelled by using AlphaFold 2 [3] and visualized using Pymol version 2.5.2 (http://www.pymol.org/). Residues encoded by exon 10 are highlighted in light green and include the pre-TM2 region and the following two-thirds of TM2. For better orientation, the head domain and the left flipper are highlighted in red and salmon, respectively. The two leucine residues, which are phenylalanines in the NCBI sequence NM_002561 [2], are indicated in magenta in the stick presentation. In addition, conserved residues involved in ATP binding as green sticks.

References:

1. Le K.T.; Paquet M.; Nouel D.; Babinski K.; Seguela P. Primary structure and expression of a naturally truncated human P2X ATP receptor subunit from brain and immune system. *FEBS Lett.* **1997**, 418**,** 195-199; https://doi.org/10.1016/s0014-5793(97)01380-x.

2. Bo X.N.; Jiang L.H.; Wilson H.L.; Kim M.; Burnstock G.; Surprenant A.; North R.A. Pharmacological and biophysical properties of the human $P2X_5$ receptor. *Mol. Pharmacol.* **2003**, 63**,** 1407-1416; https://doi.org/10.1124/mol.63.6.1407.

3. Jumper J.; Evans R.; Pritzel A.; Green T.; Figurnov M.; Ronneberger O.; Tunyasuvunakool K.; Bates R.; Zidek A.; Potapenko A.; Bridgland A.; Meyer C.; Kohl S.A.A.; Ballard A.J.; Cowie A.; Romera-Paredes B.; Nikolov S.; Jain R.; Adler J.; Back T.; Petersen S.; Reiman D.; Clancy E.; Zielinski M.; Steinegger M.; Pacholska M.; Berghammer T.; Bodenstein S.; Silver D.; Vinyals O.; Senior A.W.; Kavukcuoglu K.; Kohli P.; Hassabis D. Highly accurate protein structure prediction with AlphaFold. Nature **2021**, 596, 583-589; https://doi.org/10.1038/s41586-021-03819-2.

```
Our hP2X5FL      1 MGQAGCKGLCLSLFDYKTEKYVIAKNKKVGLLYRLLQASILAYLVVWVFLIKKGYQDVDT    60
                   MGQAGCKGLCLSLFDYKTEKYVIAKNKKVGLLYRLLQASILAYLVVWVFLIKKGYQDVDT
Q93086.4         1 MGQAGCKGLCLSLFDYKTEKYVIAKNKKVGLLYRLLQASILAYLVVWVFLIKKGYQDVDT    60

Our hP2X5FL     61 SLQSAVITKVKGVAFTNTSDLGQRIWDVADYVIPAQGENVFFVVTNLIVTPNQRQNVCAE   120
                   SLQSAVITKVKGVAFTNTSDLGQRIWDVADYVIPAQGENVFFVVTNLIVTPNQRQNVCAE
Q93086.4        61 SLQSAVITKVKGVAFTNTSDLGQRIWDVADYVIPAQGENVFFVVTNLIVTPNQRQNVCAE   120

Our hP2X5FL    121 NEGIPDGACSKDSDCHAGEAVTAGNGVKTGRCLRRENLARGTCEIFAWCPLETSSRPEEP   180
                   NEGIPDGACSKDSDCHAGEAVTAGNGVKTGRCLRRENLARGTCEIFAWCPLETSSRPEEP
Q93086.4       121 NEGIPDGACSKDSDCHAGEAVTAGNGVKTGRCLRRENLARGTCEIFAWCPLETSSRPEEP   180

Our hP2X5FL    181 FLKEAEDFTIFIKNHIRFPKFNFSKSNVMDVKDRSLLKSCHFGPKNHYCPILRLGSVIRW   240
                   FLKEAEDFTIFIKNHIRFPKFNFSKSNVMDVKDRS LKSCHFGPKNHYCPI RLGSVIRW
Q93086.4       181 FLKEAEDFTIFIKNHIRFPKFNFSKSNVMDVKDRSFLKSCHFGPKNHYCPIFRLGSVIRW   240

Our hP2X5FL    241 AGSDFQDIALEGGVIGINIEWNCDLDKAASECHPHYSFSRLDNKLSKSVSSGYNFRFARY   300
                   AGSDFQDIALEGGVIGINIEWNCDLDKAASECHPHYSFSRLDNKLSKSVSSGYNFRFARY
Q93086.4       241 AGSDFQDIALEGGVIGINIEWNCDLDKAASECHPHYSFSRLDNKLSKSVSSGYNFRFARY   300

Our hP2X5FL    301 YRDAAGVEFRTLMKAYGIRFDVMVNGKAGKFSIIPTIINVGSGVALMGAGAFFCDLVLIY   360
                   YRDAAGVEFRTLMKAYGIRFDVMVNGK                      GAFFCDLVLIY
Q93086.4       301 YRDAAGVEFRTLMKAYGIRFDVMVNGK----------------------GAFFCDLVLIY   338

Our hP2X5FL    361 LIKKREFYRDKKYEEVRGLEDSSQEAEDEASGLGLSEQLTSGPGLLGMPEQQELQEPPEA   420
                   LIKKREFYRDKKYEEVRGLEDSSQEAEDEASGLGLSEQLTSGPGLLGMPEQQELQEPPEA
Q93086.4       339 LIKKREFYRDKKYEEVRGLEDSSQEAEDEASGLGLSEQLTSGPGLLGMPEQQELQEPPEA   398

Our hP2X5FL    421 KRGSSSQKGNGSVCPQLLEPHRST  444
                   KRGSSSQKGNGSVCPQLLEPHRST
Q93086.4       399 KRGSSSQKGNGSVCPQLLEPHRST  422
```

Schiller et al. Suppl. Fig. 1

Figure S1. Amino acid sequence of the hP2X5^{FL} subunit compared with best hits in the NCBI protein database.

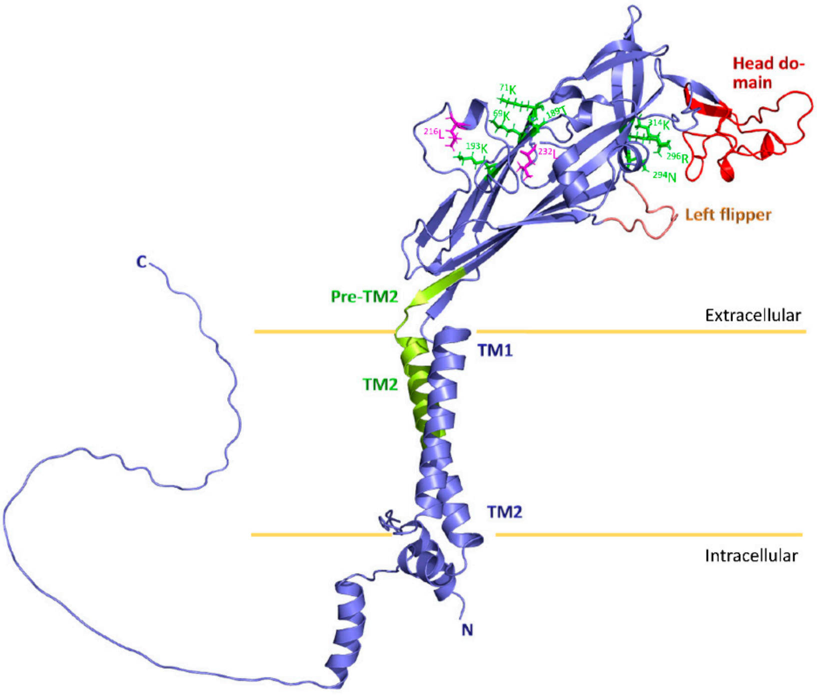

Schiller et al. Suppl. Fig. 2

Figure S2. 3D structure of a hP2X5^{FL} protomer modelled with AlphaFold 2. .
